# Light-induced rotations of chiral birefringent microparticles in optical tweezers

**DOI:** 10.1038/srep31977

**Published:** 2016-09-07

**Authors:** M. G. Donato, A. Mazzulla, P. Pagliusi, A. Magazzù, R. J. Hernandez, C. Provenzano, P. G. Gucciardi, O. M. Maragò, G. Cipparrone

**Affiliations:** 1 CNR-IPCF, Istituto per i Processi Chimico-Fisici, V. le F. Stagno D’Alcontres 37, 98158 Messina, Italy; 2 CNR- Nanotec, UOS Cosenza, Ponte P. Bucci, Cubo 33B, 87036 Rende (CS), Italy; 3 Dipartimento di Fisica, Università della Calabria, Ponte P. Bucci, Cubo 33B, 87036 Rende (CS), Italy

## Abstract

We study the rotational dynamics of solid chiral and birefringent microparticles induced by elliptically polarized laser light in optical tweezers. We find that both reflection of left circularly polarized light and residual linear retardance affect the particle dynamics. The degree of ellipticity of laser light needed to induce rotations is found. The experimental results are compared with analytical calculations of the transfer of angular moment from elliptically polarized light to chiral birefringent particles.

Optical tweezers^1^,^2^ (OT) are a powerful tool to study optical forces and torques on physical systems both at the micro^3^,^4^,^5^,^6^,^7^ and the nanoscale^8^,^9^. Optical forces are the consequence of the conservation of linear momentum in the light-matter interaction, driving particles to a laser beam focal spot if their index of refraction is higher than that of the surrounding medium^2^. Angular momentum (AM) may be transferred as well, inducing optical reaction torques on trapped objects^10^,^11^. Particle alignment and/or rotations in OT have been observed as a consequence of anisotropic scattering of trapping light on non-spherical particles^12^,^13^,^14^, of light absorption^15^,^16^,^17^,^18^ or birefringence^3^,^19^ by dielectric particles due to the interaction with beams carrying spin and/or orbital AM^20^.

Chirality derives from the lack of mirror symmetry of an object. The two specular images of the object cannot be superimposed, and are defined as the left- or right-handed version (enantiomer) of the object itself. Circularly polarized (CP) light is also a chiral entity, where the left or right handedness depends on the electric field sense of rotation. The interaction of chiral light with chiral objects, such as cholesteric liquid crystals (CLCs), gives rise to the selective circular reflection^21^,^22^,^23^, which can be used to study the transfer of both linear and angular momentum from light beams to matter^23^,^24^,^25^,^26^. In particular, control of radiation pressure^24^, passive optical sorting^25^, helicity-dependent optical trapping^26^ of spherical liquid crystal droplets, and trapping^21^,^22^ and rotations of solid chiral microparticles^23^,^27^ have been observed.

Here, we investigate the dependence of rotational frequency of chiral birefringent microparticle on the ellipticity of the incident light. The expected sinusoidal dependence of chiral particles rotation related only to the selective Bragg reflection, as reported in the ref. 23 is not sufficient to explain observations in the case of microparticles in the low chirality regime^28^. An additional contribution to the optical torque due to the optical retardance of the microparticles needs to be considered to fit our experimental data. In the first part of the paper we calculate the radiation torque on a chiral birefringent particle due to the interaction with a plane electromagnetic wave. In the second part we compare the theory with the experimental results by fitting the observed rotational frequency dependence with light ellipticity. Finally, we discuss the different components of the optical torque acting on the chiral birefringent particles.

## Results

### Calculation of the torque due to chiral reflection and optical retardance

The selective Bragg reflection of left circularly polarized (LCP) light causes the transfer of AM from a trapping beam to a left-chiral microparticle in an optical tweeers^23^, with a transfer of optical torque *2R*^+^*P/ω*, *R*^+^ being the reflectance of the material to LCP light. On the contrary, right circularly polarized (RCP) light is fully transmitted and it does not contribute to optical torque. In the general case of elliptically polarized light, the transferred optical torque upon reflection on chiral structures takes the form (*P/ω*) *R*^+^(*1* + sin *2φ*) where *φ* is the degree of ellipticity of the light (*φ* = 0 or *π/2* corresponds to linearly polarized beam, *φ* = ±*π/4* to circularly polarized light). Under the effect of this torque, particles may rotate if the selective Bragg reflection is sufficiently large^23^. Thus, the particle rotation frequency *f* is obtained by the equilibrium condition between optical torque and viscous drag torque *Γ*_*drag*_ = *8πηR*_*0*_^*3*^*Ω*, where *η* is the viscosity of the surrounding medium, *R*_*0*_ is the particle radius and *Ω* = *2π f* is the angular rotation frequency.

We consider an elliptically polarized field incident on a chiral birefringent particle in low chirality regime where a residual retardance can affect the optomechanical behaviour. This field is expressed in terms of components parallel and perpendicular to the optical axis of the material^3^,^29^ (see also Supplementary Information):


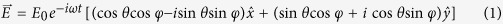


where *φ* is the ellipticity of the field and *θ* is the angle formed by the axis of the waveplate yielding the light ellipticity and the optical axis of the material. The plane wave spin angular momentum (SAM) density^2^ is:


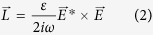


We model the light-particle interaction as a two-step phenomenon: first the light is partially transmitted by the chiral particle with Fresnel coefficients *t*^+^, for the LCP component, and *t*^*−*^, for the RCP one, respectively. Then, the transmitted light field propagates in a birefringent medium, where the phase shift due to the thickness *d* is *kdn*, with *k* being the vacuum wavevector and *n* the index of refraction. In low chirality regime^23^, we have a partial reflection of the left-handed circular component and total transmission of right-handed component, so that 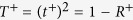, and *T*^−^ = 1. Thus, the resulting radiation torque on the birefringent left chiral particle is:


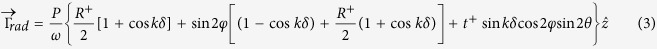


where *δ* = *d*(*n*_*e*_ − *n*_*o*_) is the residual retardance related to the material birefringence, i.e., the extraordinary, *n*_*e*_, and ordinary, *n*_*o*_, refractive indexes difference, Δ*n* = *n*_*e*_ − *n*_*o*_. We integrated on a volume element *AcΔt*, and considered that 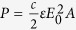 is the power of the incident light field.

### Spinning torque, alignment torque, and rotational frequency f

The radiation torque Γ_*rad*_ (Eq. 3) is the sum of three contributions (see Supplementary Information). The first and the second terms are “spinning torques” that cause the particle to spin continuously. The first is only related to the particle reflectivity, while the second one also depends on the ellipticity of the light *φ*. The third contribution is an “aligning torque” which tends to orient the optical axis of the particle with the major axis of the polarization ellipse. The total spinning torque is maximum (positive) when *φ* = *π/4* and is minimum (negative) for *φ* = *−π/4*, where the aligning torque vanishes. In general, the particle rotates as soon as the spinning part of the electromagnetic torque is greater than the aligning torque.

As discussed above, Γ_*rad*_ is the radiation torque on the chiral particle due to the light field. At equilibrium, this torque is equal to the viscous drag torque, Γ_*drag*_ = *8πηR*_*0*_^*3*^*Ω*, experienced by the rotating particle in water, with *η* = 1.002 mPa s at 20 °C. Since the angular rotation frequency is 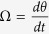, we obtain a differential equation in the variable *θ*, of the form:


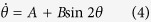


By integration^3^,^29^,^30^, the particle rotation frequency, *f*, is obtained as:


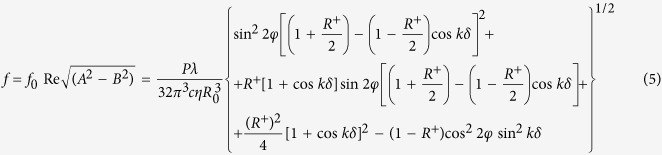


with parameters *A* and *B* proportional to the spinning torque and alignment torque, respectively (see Supplementary Information). This formula has been used to fit the dependence of measured rotation frequencies as a function of the different degree of light ellipticity *φ* in our optical tweezers experiments.

### Chiral microparticles and rotation measurements in optical tweezers

**Figure 1:**
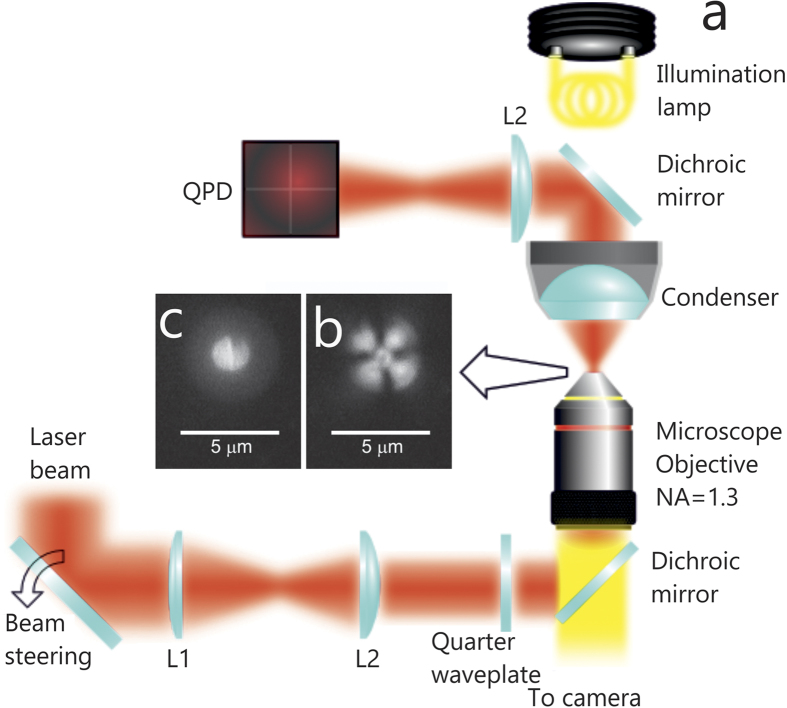
Sketch of the experimental set-up and chiral particle images. (**a**) A laser beam is expanded by a two lens telescope (L1 and L2) to overfill the back aperture of the high numerical aperture objective. Light polarization is controlled by a quarter waveplate. Light scattered from the trapped particle interferes with unscattered laser light in the back focal plane of the condenser of the microscope. The interference pattern is projected by a collection lens onto a four-quadrant photodiode (QPD). The analog outputs from each quadrants are combined to generate signals proportional to the spatial displacements *x, y, z* of the trapped particle. (**b,c**) Optical polarization microscopy images of a chiral microparticle taken in transmission (**b**) and reflection (**c**) mode through crossed polarizers.

Optical trapping and light-induced rotation measurements have been carried out on solid left(L)-chiral microparticles (Fig. 1) obtained by UV light curing of CLC droplets in two OT experimental setups (see Methods) operating at 785 nm and 830 nm wavelengths. In brief, the L-chiral microparticles we use in our studies are made of polymeric CLC, that is a chiral optically anisotropic dielectric medium in which the average molecular orientation, described by a unit vector **n** (director), rotates by 2π around the axis of the supramolecular helical structure over a distance *p*, named cholesteric pitch^31^. Such a chiral ordering may be right(R)- or left(L)-handed. Thus, due to the combination of the birefringence and the helicoidal structure, the CLC is a self-organized one dimensional photonic bandgap material, in which a wavelength band is selectively reflected due to the Bragg reflection (Fig. 1c) when light propagates along the helicoidal axis^21^,^32^. The propagation of a plane wave along this axis is forbidden for circularly polarized light with helicity parallel to the one of the material structure and wavelength within a spectral range (named *stop-band*). In our case the stop-band is located between 780 nm and 850 nm, thus, both trapping wavelengths fall within the stop-band of the chiral microparticles fulfilling the Bragg reflection condition^23^. The shell structure of the particles is determined by droplets radius, *R*_*0*_, helical pitch, *p*, and material parameters. In the particular case of particles made of a small number of pitches, a not negligible optical retardance can result when the supramolecular helix is not complete (*R*_0_/*p* is not an integer).

**Figure 2:**
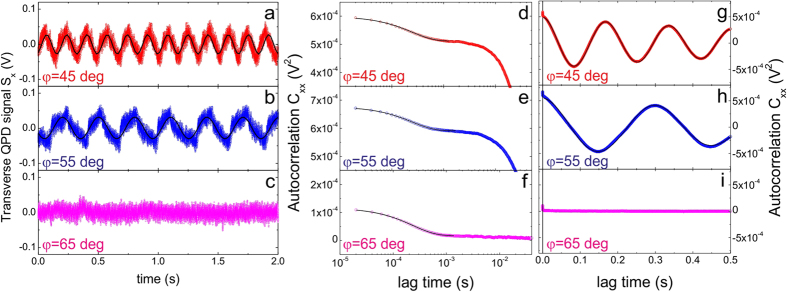
Transverse QPD signals at different waveplate angles: 45 deg (**a**), 55 deg (**b**), and 65 deg (**c**). Black lines are cosinusoidal fit to the data. In proximity of the left-handed polarization a modulation appears on top of the particle positional fluctuations typical for a trapped particle in optical tweezers indicating the onset of rotations. Autocorrelation functions (ACFs) showing the particle dynamics at short times (**d–f**) and at long times (**g–i**). At short times, the exponential decay is dictated by the optical tweezers trapping potential and the overdamped thermal fluctuations. At long times, the particle rotation appears as a cosinusoidal modulation in the ACFs when the ellipticity is close to the left-handed polarization (**g,h**), while no modulation is visible (no rotation) when the ellipticity is away from left-handed polarization.

The rotational frequencies of trapped microparticles at different light ellipticity are obtained by fitting the autocorrelation functions (ACFs) of the tracking signals acquired by back focal plane interferometry^14^,^23^ on a quadrant photodiode (QPD). Rotations of the trapped particles about the laser beam propagation direction are manifested in the transverse fluctuating QPD signals. Figure 2a,b, and c show the QPD transverse signals for an optically trapped L-chiral microparticle at different waveplate angles. The noise-like signals are related to the thermal fluctuations of the particles in the optical tweezers three-dimensional confining potential^33^. In addition to the exponential decay (Fig. 2d–f) of the positional fluctuations dictated by the OT, when particles rotate in the trap, a modulation (Fig. 2a,b) is superposed to the thermal fluctuations and a corresponding cosinusoidal oscillation appears in the transverse ACFs (Fig. 2g,h). The fitting of the exponential decay and the cosinusoidal oscillation enables an accurate measure of both the optical trapping forces and rotational frequencies for the ellipticity values that drive the particle rotations. Instead, no modulation is found otherwise (Fig. 2i).

**Figure 3:**
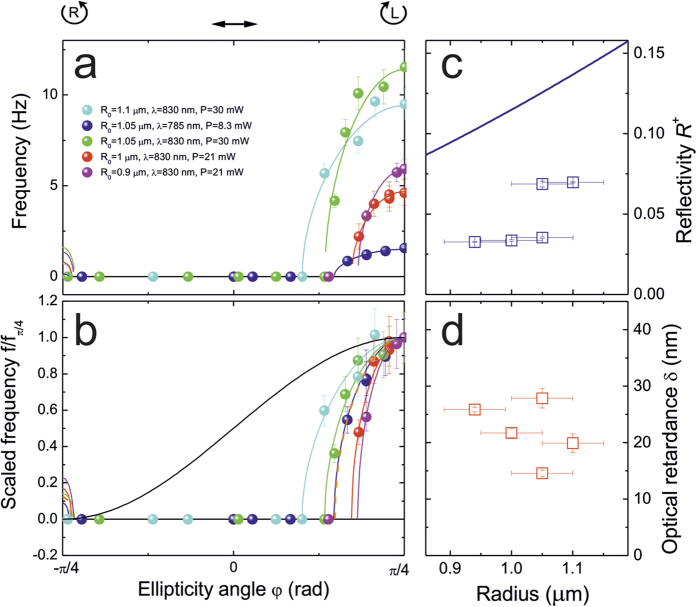
(**a**) Rotational frequencies of trapped particles as a function of the field ellipticity (angle *φ*) as measured on chiral microparticles of different size, for different power and wavelength, as summarized in the legend. Error bars represent the standard deviation from the mean value from the analysis of 3 set of transverse ACFs signals in the same experimental conditions. Continuous lines are best fit to the data following Eq. 5. (**b**) Frequency data scaled with respect to their maximum and compared with the expected behaviour if only reflection was the cause of the induced optical torque (black line). Reflectivity (**c**) and optical retardance (**d**) values and uncertainties obtained from the fit of the measured frequencies in (**a**) as a function of particle radius. Error bars on the radii represent an uncertainty of about 5% for the trapped particle size measurement from CCD images.

The results of the rotational frequency of about five sets of different microparticles with similar radius, *R*_*0*_*≈*1 μm, as a function of ellipticity are summarized in Fig. 3a. The data are arranged according to the same experimental conditions of power and wavelength (see legend in Fig. 3a). Continuous lines are best fit to the data following Eq. 5, while error bars represent the standard error from the mean value from the analysis of 3 different transverse ACFs signals. We find that rotations are observed only in a restricted waveplate angle range around left circular polarization of the trapping beam. Figure 3b shows the frequency data (and fit) normalized to their maximum (at *φ* = *π/4*) and compared with the expected behaviour if only reflection was the cause of the induced optical torque (black line). Thus, a simple (*1* + sin *2φ*) dependence due to the reflection of left-circular polarization (black curve in Fig. 3b) is not sufficient to explain the observed dependence of the rotational frequency on light ellipticity.

Indeed, it is worth noting that confining liquid crystals in micrometer-sized geometries allows a wide range of thermodynamically stable supramolecular configurations that result by the combination of chirality, elasticity, and interface properties. The self-organized internal configuration of the droplet strongly depends on the molecular anchoring at the interface and on its radius *R*_*0*_ with respect to the nominal pitch *p* of the helical structures. In our case, *R*_*0*_ ≅ 1 μm and *p* ≅ 0.51 μm, and, thus, the low chirality regime can be considered^28^,^31^,^32^ that is in agreement with a low reflectivity and a not negligible residual retardance.

The fit of all data (solid lines in Fig. 3a,b), based on Eq. 5, has been obtained with only two parameters, one connected to the reflectance, *R*^+^, of the trapped particle and the other to its optical retardance, *δ*. The fitted reflectance values range from 0.03 to 0.07 (Fig. 3c) while the residual retardance has an average of *δ* ≅ 22 nm (Fig. 3d), which corresponds to an effective birefringent layer thickness *d* of approximately 160 nm assuming the CLC nominal birefringence Δn ≅ 0.14 at the OT wavelength. Note that while for the reflectance (Fig. 3c) we can observe an increasing trend with particle radius, the optical retardance (Fig. 3d) does not appear to change in the range of particle size investigated. The reflectance values are compared with the theoretical reflectance (blue line in Fig. 3c) evaluated for a CLC layer with a thickness *d* = (*2/3*)*R*_*0*_^23^. The measured values appear to be smaller than the theoretical reflectance, suggesting that the real reflectance connected to the helical pitch is strongly affected by the distortion of the cholesteric layer when confined in a small microsized volume^28^.

**Figure 4:**
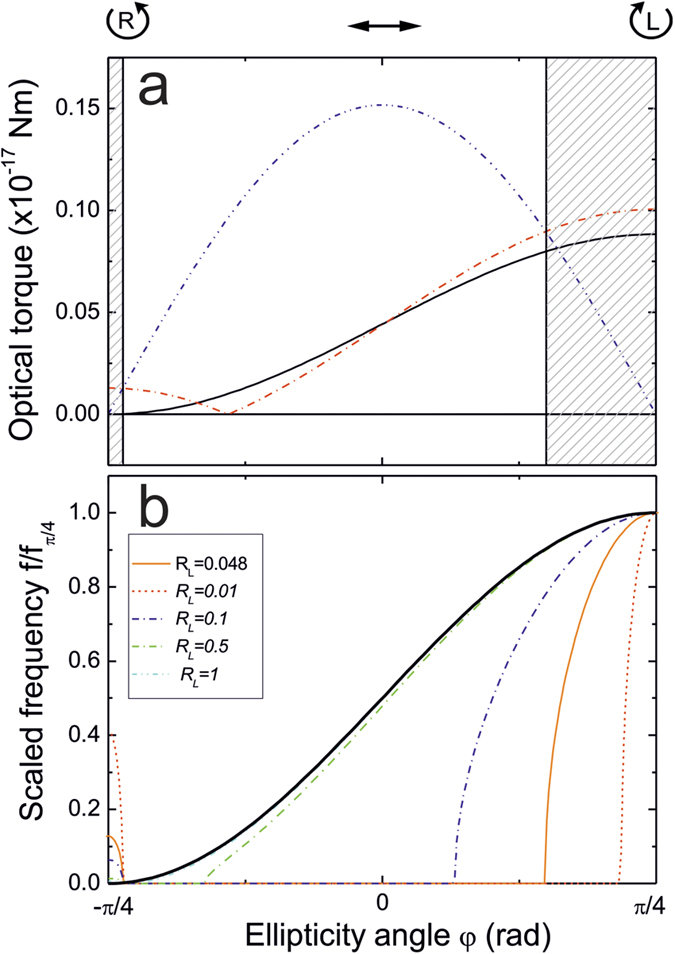
(**a**) Spinning torque (red dash-dot curve) and maximum aligning torque (blue dash-dot-dot curve) based on the averaged results of the fit in Fig. 3. Black solid curve represents the expected torque if only reflection from the chiral particle was the origin of rotations. Gray dashed areas are the regions where particle rotations are allowed. (**b**) Expected values of the rotational frequencies normalized to their maxima at different reflectance of the trapped particle and at fixed retardance (obtained as the average value from the fit of experimental data).

Equation 5 has real values, corresponding to rotations, only if *|A|* > *|B|*, that is, when the absolute value of the spinning torque is greater than the absolute value of the aligning torque (Fig. 4a). We find that rotations are possible only in a restricted range of λ/4 waveplate angles centred on LCP. The model based on Eq. 5 permits, in principle, also low frequency rotations around RCP (see fits in Fig. 3a,b), but the expected frequencies are so small that are below the detectable level related to the finite observation time (2 s) and the presence of the intrinsic thermal fluctuations in the OT.

## Discussion

The data shown in Fig. 3 and the fit based on Eq. 5 show that particle dynamics can be explained only by introducing a retardance of the trapped particle. In Fig. 4b, the expected rotational frequency at increasing reflectance coefficients and fixed retardance shows that a reflectance of *R*^+^ = 0.5 is enough to recover the simple (*1*+*sin 2φ*) behaviour. However, in experiments, the three-dimensional trapping of chiral microparticles with large reflectance is not occurring, as radiation pressure destabilizes the trap pushing the particles along the optical axis^23^. We also expect the particle residual retardance to be small, as the microparticles have a radial arrangement of the cholesteric layers and thus, are optically isotropic^22^.

In conclusion, we have reported on the transfer of AM from elliptically polarized trapping beams to chiral birefringent microparticles. The model we used, which takes into account the reflectivity of chiral mirrors and a residual retardance effect of the material, fits well the experimental observations with microparticles in low chirality regime. We have also found the angular regimes in which the ellipticity of the trapping beams can induce rotations. This unique combination of chiral/birefringent optomechanical properties paves the way to a wider range of optical control of the mechanical interaction between light and matter at the mesoscale.

## Methods

Chiral microparticles^21^ have an inner structure based on L-chiral cholesteric concentric layers in a spherulite-like radial configuration. The reflectance and transmittance of these microparticles can be finely tuned by controlling the particle size (*i.e.*, particle radius *R*_*0*_). They are prepared as follows. First, a pure reactive mesogen contained in RMS03-001C blend (Merck, Germany) is obtained extracting its solvent PGMEA (propylene glycol monomethyl ether acetate) by vacuum evaporation at 90 Celsius. This is mixed with 14 wt% L-chiral dopant (ZLI-811, Merck) yielding a CLC phase. This has a photonic band gap centred at about 815 nm, fulfilling the selective reflection condition for both the wavelengths (830 and 785 nm) used in our optical tweezers. Then, a CLC droplet emulsion is prepared with type I ultrapure water. The emulsion is polymerized in nitrogen environment for at least 4 h under a UV lamp having emission wavelength centred at about 350 nm, obtaining solid chiral particles with size ranging from hundreds of nanometres up to tens of microns. The refractive indexes, *n*_*e*_ = 1.66 and *n*_*o*_ = 1.52, of the chiral material are evaluated by the Cauchy formula for our wavelengths^23^, resulting in an helicoidal pitch, *p*, of approximately 0.51 μm.

Few tens of microliters of a chiral particle aqueous suspension are loaded in a small glass chamber for optical trapping experiments. Two different optical tweezers setups are used with diode laser sources at 830 nm (150 mW) and 785 nm (80 mW) wavelength determined from specsheets. The diode lasers are home-built and stabilized in temperature and current through active feedback circuits. High numerical aperture objectives (Olympus Uplan FLN, X100, NA = 1.3) give diffraction limited spots. Optical power at the sample is measured by a power meter (Thorlabs PM160) placed at the output pupil of the objective lens. Its measure has an uncertainty of about 10% estimated as the standard error over five successive measurements. The ellipticity of light is controlled with both liquid-crystals (Thorlabs LCC1111-B Liquid Crystal Retarder driven by Thorlabs LCC25 Lyquid Crystal Controller) and solid λ/4 and λ/2 waveplates. Back focal plane interferometry^33^, where the interference between the laser beam and the light scattered by the trapped particle is imaged on a four-quadrant photodiode, is used to detect the trapped particle rotations^14^. The autocorrelation function of the tracking signals (Fig. 2) is analysed to obtain the particle rotation frequencies^23^. In fact, for a rotating particle in an optical trap, ACFs are^14^:


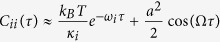


with 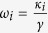 the relaxation frequency, *κ*_*i*_ the trap spring constant in the *i*-direction, *γ* the drag coefficient, *a* the calibration amplitude of the particle rotation and *Ω* the angular rotation frequency. Thus, from the fit of the experimental ACFs, both trap spring constants and rotational frequency can be obtained, provided that *γ* = 6π*R*_*0*_*η*, with *η* the medium viscosity, is known. To this aim, the radius *R*_*0*_ of each trapped particle is measured by video microscopy. To monitor the trapped particles, a charge coupled device camera is used (Fig. 1a). The trapped particle images are calibrated by imaging a microscope slide ruler, with an estimated uncertainty Δ*R* of 0.05 μm related to the image resolution for visible light. For each particle image the diameter is measured 5 times from the CCD image, so that a mean radius, *R*_*0*_, is obtained with an estimated uncertainty of about 5%.

## Additional Information

**How to cite this article**: Donato, M. G. *et al.* Light-induced rotations of chiral birefringent microparticles in optical tweezers. *Sci. Rep.* **6**, 31977; doi: 10.1038/srep31977 (2016).

## Supplementary Material

Supplementary Information
