# Supplementary material for: Light-induced rotations of chiral birefringent microparticles in optical tweezers
Source: Sci Rep. 2016 Sep 7;6:31977. doi: 10.1038/srep31977 (PMC5013277; doi:10.1038/srep31977)
Supplement: Supplementary Information [file srep31977-s1.pdf]

Supplementary Information

Light-induced rotations of chiral birefringent microparticles in optical tweezers

M. G. Donato[1], A. Mazzulla[2], P. Pagliusi[2,3], A. Magazzù[1], J. R. Hernandez[3], C. Provenzano[3], P. G. Gucciardi[1], O. M. Maragò[1,*], and G. Cipparrone[2,3,*]

[1]CNR-IPCF, Istituto per i Processi Chimico-Fisici, V. le F. Stagno D'Alcontres 37, 98158 Messina, Italy, [2]CNR-Nanotec, UOS Cosenza, Ponte P. Bucci, Cubo 33B, 87036 Rende (CS), Italy, [3]Dipartimento di Fisica, Università della Calabria, Ponte P. Bucci, Cubo 33B, 87036 Rende (CS), Italy.

S.1 Calculation of the torque considering chiral reflection and optical retardance.

We consider an elliptically polarized field incident on a particle:

$$\vec{E}_{in} = E_0 e^{-i\omega t}\left(\cos\varphi\hat{x} + i\sin\varphi\hat{y}\right)$$

To express the dependence of the field from the angle θ, which is the angle formed by the axis of the waveplate giving the ellipticity and the optical axis of the material, we first use the rotation matrix *R(θ):*

$$\begin{pmatrix} E_x \\ E_y \end{pmatrix} = \begin{pmatrix} \cos\theta & -\sin\theta \\ \sin\theta & \cos\theta \end{pmatrix}\begin{pmatrix} \cos\varphi \\ i\sin\varphi \end{pmatrix}$$

giving

$$\vec{E} = E_0 e^{-i\omega t}\left[\left(\cos\theta\cos\varphi - i\sin\theta\sin\varphi\right)\hat{x} + \left(\sin\theta\cos\varphi + i\cos\theta\sin\varphi\right)\hat{y}\right]$$

Now, let us consider the circular basis $\left(\hat{e}_+, \hat{e}_-, \hat{z}\right)$ for which $\hat{x} = \frac{1}{\sqrt{2}}\left(\hat{e}_+ + \hat{e}_-\right)$ and $\hat{y} = -i\frac{1}{\sqrt{2}}\left(\hat{e}_+ - \hat{e}_-\right)$. The transmitted field will be:

$$\vec{E}_{Tr} = \frac{E_0}{\sqrt{2}} e^{-i\omega t}\left\{\begin{matrix} t_+\hat{e}_+\left[\left(\cos\varphi\cos\theta - i\sin\varphi\sin\theta\right) - i\left(\cos\varphi\sin\theta + i\sin\varphi\cos\theta\right)\right] + \\ +t_-\hat{e}_-\left[\left(\cos\varphi\cos\theta - i\sin\varphi\sin\theta\right) + i\left(\cos\varphi\sin\theta + i\sin\varphi\cos\theta\right)\right] \end{matrix}\right\}$$

with t_+, t_- Fresnel coefficients for left circular (LCP) and right circular (RCP) polarization, respectively. Here, we suppose that left and right circular components of the field have different transmission coefficients, due to the selective Bragg reflection shown by cholesteric liquid crystals.

The transmitted field is then expressed again in the cartesian basis:

$$\vec{E}_{Tr} = \frac{E_0}{2} e^{-i\omega t}\left\{\begin{matrix} \hat{x}\begin{bmatrix} t_+\left(\cos\theta\cos\varphi - i\sin\theta\sin\varphi\right) - it_+\left(\sin\theta\cos\varphi + i\cos\theta\sin\varphi\right) + \\ +t_-\left(\cos\theta\cos\varphi - i\sin\theta\sin\varphi\right) + it_-\left(\sin\theta\cos\varphi + i\cos\theta\sin\varphi\right) \end{bmatrix} + \\ +\hat{y}\begin{bmatrix} t_+\left(\sin\theta\cos\varphi + i\cos\theta\sin\varphi\right) + it_+\left(\cos\theta\cos\varphi - i\sin\theta\sin\varphi\right) + \\ +t_-\left(\sin\theta\cos\varphi + i\cos\theta\sin\varphi\right) - it_-\left(\cos\theta\cos\varphi - i\sin\theta\sin\varphi\right) \end{bmatrix} \end{matrix}\right\}$$

Now, we explicitly consider the birefringence. We impose (like in [1]) that the extraordinary ray is directed along the *x* axis whereas the ordinary ray is directed along *y*. The phase shift due to the crossing a thickness *d* of materal is *kdn*, where *k* is the vacuum wavevector and *n* the index of refraction:

$$\vec{E}_{Tr} = \frac{E_0}{2} e^{-i\omega t} \begin{Bmatrix} \hat{x} e^{ikdn_e} \left[(t_+ + t_-)(\cos\theta\cos\varphi - i\sin\theta\sin\varphi) - i(t_+ - t_-)(\sin\theta\cos\varphi + i\cos\theta\sin\varphi) \right] + \\ + \hat{y} e^{ikdn_o} \left[(t_+ + t_-)(\sin\theta\cos\varphi + i\cos\theta\sin\varphi) + i(t_+ - t_-)(\cos\theta\cos\varphi - i\sin\theta\sin\varphi) \right] \end{Bmatrix}$$

Its complex conjugate is:

$$\vec{E}^*_{Tr} = \frac{E_0}{2} e^{i\omega t} \begin{Bmatrix} \hat{x} e^{-ikdn_e} \left[(t_+ + t_-)(\cos\theta\cos\varphi + i\sin\theta\sin\varphi) + i(t_+ - t_-)(\sin\theta\cos\varphi - i\cos\theta\sin\varphi) \right] + \\ + \hat{y} e^{-ikdn_o} \left[(t_+ + t_-)(\sin\theta\cos\varphi - i\cos\theta\sin\varphi) - i(t_+ - t_-)(\cos\theta\cos\varphi + i\sin\theta\sin\varphi) \right] \end{Bmatrix}$$

The correspondent angular momentum density will be:

$$\vec{L}_{Tr} = \frac{\varepsilon}{2i\omega} \vec{E}^*_{Tr} \times \vec{E}_{Tr} =$$

$$= \frac{\varepsilon}{2i\omega} \frac{E_0^2}{4} \begin{Bmatrix} e^{ikd(n_o - n_e)} \left[(t_+ + t_-)(\cos\theta\cos\varphi + i\sin\theta\sin\varphi) + i(t_+ - t_-)(\sin\theta\cos\varphi - i\cos\theta\sin\varphi) \right] \cdot \\ \left[(t_+ + t_-)(\sin\theta\cos\varphi + i\cos\theta\sin\varphi) + i(t_+ - t_-)(\cos\theta\cos\varphi - i\sin\theta\sin\varphi) \right] + \\ -e^{-ikd(n_o - n_e)} \left[(t_+ + t_-)(\sin\theta\cos\varphi - i\cos\theta\sin\varphi) - i(t_+ - t_-)(\cos\theta\cos\varphi + i\sin\theta\sin\varphi) \right] \cdot \\ \left[(t_+ + t_-)(\cos\theta\cos\varphi - i\sin\theta\sin\varphi) - i(t_+ - t_-)(\sin\theta\cos\varphi + i\cos\theta\sin\varphi) \right] \end{Bmatrix} \hat{z}$$

After some lengthy calculations we will have:

$$\vec{L}_{Tr} = \frac{\varepsilon E_0^2}{4\omega} \left\{ (T^+ - T^-)\cos k\delta + (T^+ + T^-)\cos k\delta \sin 2\varphi - 2t_+t_- \sin k\delta \cos 2\varphi \sin 2\theta \right\} \hat{z}$$

where $\delta = d(n_e - n_0)$ is the optical retardance and $t_+^2 = T^+ = 1 - R^+$ and $t_-^2 = T^-$ are the transmittances of the material for the LCP and RCP components of the field. The density of angular momentum due to the reflected beam is [2]

$$\vec{L}_{\mathrm{Re}\,fl} = -\frac{\varepsilon E_0^2}{4\omega} R^+ (1 + \sin 2\varphi) \hat{z}$$

The density of angular momentum due to the incident field is:

$$\vec{L}_{in} = \frac{\varepsilon E_0^2}{2\omega} \sin 2\varphi \hat{z}$$

Thus, the density of the total reaction torque is:

$$\begin{aligned} \Delta\vec{L} &= \vec{L}_{in} - \vec{L}_{out} = \vec{L}_{in} - \left(\vec{L}_{Tr} + \vec{L}_{\mathrm{Re}\,fl} \right) = \\ &= \frac{\varepsilon E_0^2}{4\omega} \left\{ 2\sin 2\varphi + R^+(1 + \sin 2\varphi) - (T^+ - T^-)\cos k\delta - (T^+ + T^-)\cos k\delta \sin 2\varphi + 2t_+t_- \sin k\delta \cos 2\varphi \sin 2\theta \right\} \hat{z} \end{aligned} \quad (1)$$

If R^+=0 and T^- =1, the Friese et al. [1] formula for the density of angular momentum is obtained:

$$\Delta\vec{L}(R^+ = 0,\ T^- = 1) = \frac{\varepsilon E_0^2}{2\omega} \left\{ \sin 2\varphi \left[1 - \cos kd(n_o - n_e) \right] - \sin kd(n_o - n_e) \cos 2\varphi \sin 2\theta \right\} \hat{z}$$

Furthermore, in absence of retardance (δ=*0*) and T^- =1, we obtain:

$$\Delta\vec{L} = \frac{\varepsilon E_0^2}{2\omega} \left[R^+ (1 + \sin 2\varphi) \right] \hat{z}$$

that, by integrating on a volume element $Ac\Delta t$ and taking in account that $P = \frac{c}{2}\varepsilon E_0^2 A$ gives [2]:

$$\vec{\Gamma}_{Rad} = \frac{P}{\omega}\left[R^+\left(1+\sin 2\varphi\right)\right]\hat{z}$$

Alignment torque and Spinning torque.

By applying this same integration to equation (1) we obtain:

$$\vec{\Gamma}_{Rad} = \frac{P}{2\omega}\begin{bmatrix} 2\sin 2\varphi - \left(T^+ - T^-\right)\cos k\delta - \left(T^+ + T^-\right)\sin 2\varphi\cos k\delta + \\ +2t_+t_-\sin k\delta\cos 2\varphi\sin 2\theta + R^+\left(1+\sin 2\varphi\right) \end{bmatrix}\hat{z} \qquad (2)$$

If T^+=1- R^+ and T^- =1 (left-circularly polarized component is reflected with reflectance R^+, right-circularly polarized component of light is completely transmitted), we obtain

$$\vec{\Gamma}_{Rad} = \frac{P}{\omega}\left\{\begin{matrix} \frac{R^+}{2}\left[1+\cos k\delta\right]+ \\ +\sin 2\varphi\left[\left(1-\cos k\delta\right)+\frac{R^+}{2}\left(1+\cos k\delta\right)\right]+ \\ +t_+\sin k\delta\cos 2\varphi\sin 2\theta \end{matrix}\right\}\hat{z} \qquad (3)$$

Equation (3) is the sum of three contributions. The first and the second terms are “spinning torques” that cause the particle to spin continuously. The first is only related to the particle reflectivity, while the second one also depends on the ellipticity of the light φ. The third contribution is an “aligning torque” which tends to orient the optical axis of the particle with the major axis of the polarization ellipse. The total spinning torque is maximum (positive) when $\varphi=\pi/4$ and is minimum (negative) for $\varphi=-\pi/4$, where the aligning torque vanishes. In general, the particle rotates as soon as the spinning part of the electromagnetic torque is greater than the aligning torque.

Fitting function for the rotational frequency *f*

As above discussed, equation (2) is the reaction torque on the chiral particle due to the light field. At equilibrium, this torque will be equal to the viscous drag torque:

$$\frac{P}{2\omega}\left[2\sin 2\varphi - \left(T^+ - T^-\right)\cos k\delta - \left(T^+ + T^-\right)\sin 2\varphi\cos k\delta + 2t_+t_-\sin k\delta\cos 2\varphi\sin 2\theta + R^+\left(1+\sin 2\varphi\right)\right] = 8\pi\eta R_0^3\Omega \qquad (4)$$

where η is the water viscosity (at 20°C, 1.002 mPa s). As $\Omega = \frac{d\theta}{dt}$, equation (4) is a differential equation in the variable θ, in the form

$$\dot{\theta} = A + B\sin 2\theta$$

As Friese et al. [1] and Manzo et al. [3] noted, the frequency of rotation will be

$$\Omega = \Omega_0 \operatorname{Re}\left[\sqrt{\left(A^2 - B^2\right)}\right] \qquad (5)$$

In our case,

$$A = \frac{P}{16\pi\omega\eta R_0^3}\left\{2\sin 2\varphi + R^+\left(1+\sin 2\varphi\right) - \left(T^+ - T^-\right)\cos k\delta - \left(T^+ + T^-\right)\cos k\delta \sin 2\varphi\right\}$$

and

$$B = \frac{P}{16\pi\omega\eta R_0^3}\left\{2t_+t_- \sin k\delta \cos 2\varphi\right\}$$

Considering T_R=1, we obtain

$$A = \frac{P}{8\pi\omega\eta R_0^3}\left\{\sin 2\varphi\left[\left(1+\frac{R^+}{2}\right) - \left(1-\frac{R^+}{2}\right)\cos k\delta\right] + \frac{R^+}{2}\left(1+\cos k\delta\right)\right\} \quad (6)$$

and

$$B = \frac{P}{8\pi\omega\eta R_0^3}\left\{t_+ \sin k\delta \cos 2\varphi\right\} \quad (7)$$

It is worth noting A and B are proportional to the spinning torque and to the alignment torque the particle is subjected to. By substituting the formulas for A and B in equation (5) we will have

$$f = \frac{P\lambda}{32\pi^3 c\eta R_0^3}\left\{\begin{array}{l} \sin^2 2\varphi\left[\left(1+\frac{R^+}{2}\right) - \left(1-\frac{R^+}{2}\right)\cos k\delta\right]^2 + \\ +R^+\left[1+\cos k\delta\right]\sin 2\varphi\left[\left(1+\frac{R^+}{2}\right) - \left(1-\frac{R^+}{2}\right)\cos k\delta\right] + \\ +\frac{\left(R^+\right)^2}{4}\left[1+\cos k\delta\right]^2 - \left(1-R^+\right)\cos^2 2\varphi \sin^2 k\delta \end{array}\right\}^{1/2} \quad (8)$$

as $\omega = \frac{2\pi c}{\lambda}$ and $f = \frac{\Omega}{2\pi}$. This equation has been used to fit the dependence of measured rotation frequencies on the different ellipticity angle φ of light.

References

[1] Friese M. E. J., Nieminen T. A., Heckenberg N. R., Rubinsztein-Dunlop H. Optical alignment and spinning of laser-trapped microscopic particles. *Nature* **394**, 348-350 (1998).

[2] Donato M. G., et al. Polarization-dependent optomechanics mediated by chiral microresonators, *Nat. Commun.* **5**, 3656 (2014).

[3] Manzo C., Paparo D., Marrucci L., Janossy I. Light-induced rotation of dye-doped liquid crystal droplets. *Phys. Rev. E* **73**, 051707 (2006)
